# Obstructive accessory mitral valve tissue in an adult patient: a case report

**DOI:** 10.1186/s13256-019-2110-z

**Published:** 2019-06-17

**Authors:** Mahmoud Mardenli, Abdulla Samman, Hussein Alkanj, Amal Babi

**Affiliations:** 1 Cardiology Department, Aleppo University Hospital, Aleppo, Syria; 2 Cardiac Surgery Department, Aleppo University Hospital, Aleppo, Syria; 3 Echocardiography Department, Aleppo University Hospital, Aleppo, Syria

**Keywords:** Accessory mitral valve tissue, Left ventricular outflow tract obstruction, Echocardiography

## Abstract

**Background:**

Accessory mitral valve tissue is a rare congenital anomaly that is commonly diagnosed in early childhood and rarely in adulthood. It is usually asymptomatic. However, it may cause left ventricular outflow tract obstruction in a way that mimics various other causes of obstruction.

**Case summary:**

A 72-year-old Caucasian man complained of chest discomfort and exertional dyspnea for 3 months. There were no specific findings from a physical examination except systolic murmur. Transthoracic echocardiography demonstrated a mass on the mitral valve extending to the intraventricular septal, raising the pressure gradient flow across the aortic valve. Transesophageal echocardiography showed parachute-like tissue connected to the anterior leaflet of the mitral valve causing left ventricular outflow tract obstruction. During the surgery preparation period, he underwent coronary angiography and computed tomography to study the anatomy surrounding the mass. After surgery, biopsy showed non-specific findings.

**Conclusion:**

When facing a case of aortic valve stenosis, accessory mitral valve tissue should be kept in mind as one of the possible underlying causes despite its rarity. Although it is simple and noninvasive, echocardiography remains the best diagnostic procedure to make the correct decision about management and to define the golden time for surgical intervention.

**Electronic supplementary material:**

The online version of this article (https://doi.org/10.1186/s13256-019-2110-z) contains supplementary material, which is available to authorized users.

## Background

Accessory mitral valve tissue (AMVT) is a rare congenital cardiac malformation that sometimes causes left ventricular outflow tract (LVOT) obstruction and is commonly associated with other congenital cardiac anomalies [1]. It is usually present in childhood causing LVOT obstruction. Such an obstruction leads to chest pain, effort syncope, or recurrent transient ischemic attack/stroke. The number of adults affected by such a disease is one per 26,000 echocardiograms [2].

The first case of this rare condition was described in the literature in 1842 [3], whereas the first surgical treatment was described in 1963 [4]. The majority of patients who were reported to have varying degrees of LVOT obstruction were diagnosed with AMTV during the first 10 years of their life. However, LVOT was rarely detected in adulthood [5].

Here is a case report of an adult patient with AMVT, whose disease developed a severe obstruction of LVOT.

## Case presentation

A 72-year-old Caucasian man, suffering chest pain, visited our emergency department after being diagnosed as having dyspnea. The dyspnea started 3 months ago and deteriorated the week before visiting our emergency department. His dyspnea occurred with moderate exertion without any associated symptoms. His chest pain was atypical with some parietal characteristics. He was a heavy tobacco smoker with no medical history and with no chronic medications prescribed. However, hypertension seemed to have run in his family.

A physical examination revealed severe systolic murmur in the aortic area radiating toward the left parasternal space, becoming fainter at the apex. Blood pressure was symmetric, measuring 155/75 mmHg, pulse rate was 95 beats per minute (bpm). Lungs were clear on auscultation without crackles or abnormal sounds. A chest X-ray showed normal cardiac silhouette and aortic arch, and both lungs were clear and expanded with no infiltrates or pleural effusions. An electrocardiogram showed non-specific changes with T wave inversion on lateral leads and horizontal ST segment depression on V_4–6_.

**Fig. 1:**
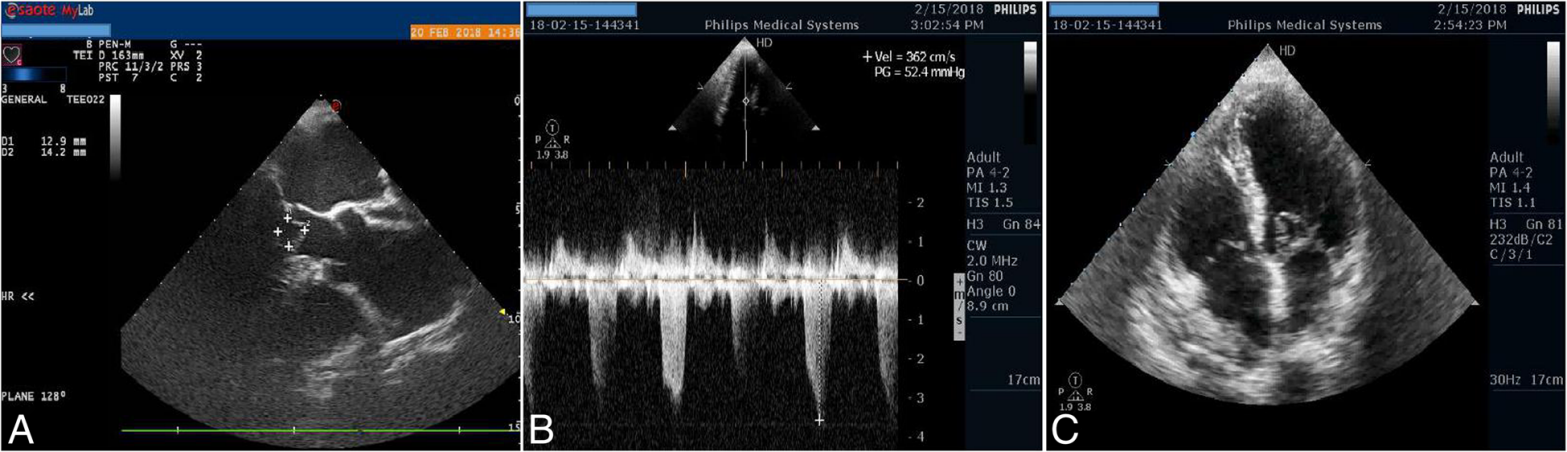
**a** Transesophageal echocardiography demonstrated tissue dimensions and location. **b** Transthoracic echocardiography pressure peak gradient measured by continuous wave Doppler across left ventricular outflow tract. **c** Transthoracic echocardiography apical four-chamber view showed the relation between the accessory mitral valve tissue and the anterior leaflet of the mitral valve

He was admitted to the coronary care unit to follow up on the process of examining his body functions. Transthoracic echocardiography (TTE) revealed an oval-like tissue with clean margins attached to the proximal portion of the anterior leaflet of the mitral valve causing LVOT occlusion during systole. The gradient pressure through LVOT measured 55 mmHg, without organic lesion in the aortic cusps. The left ventricle wall motion was normal. Dimensions at systolic and diastolic phases were normal. Pulmonary pressure was 18 mmHg. No other cardiac anomalies were present (Fig. 1b, c, Additional file 1: Video S1).

To obtain more detailed information, transesophageal echocardiography (TEE) was performed. This revealed a parachute-like structure (measuring 13 × 14 mm) attached to the proximal portion of the anterior leaflet of the mitral valve causing LVOT obstruction (Fig. 1a, Additional file 2: Video S2).

**Fig. 2:**
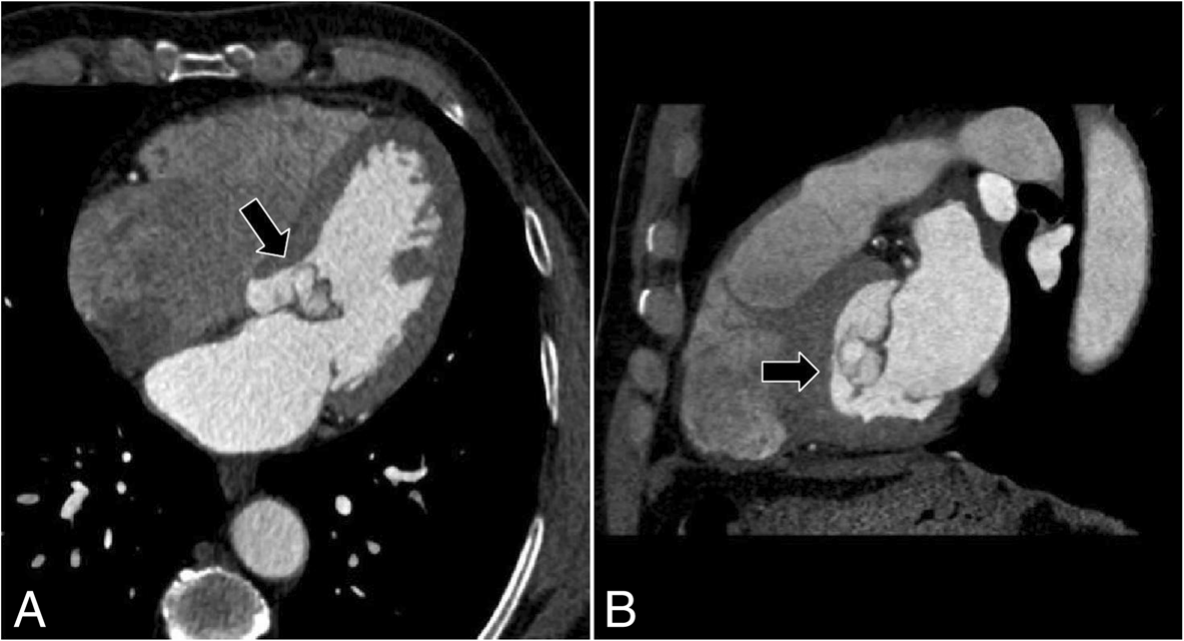
Multi-slice computed tomography. **a** Axial section demonstrated the mass. **b** Sagittal section illustrated the location of the mass and relation to aortic valve. The arrows refer to the left ventricle output where the mass was found

Metoprolol tartrate 50 mg twice daily was prescribed for our patient until the scheduled surgery. During hospitalization, multi-slice computed tomography (MS-CT) scan was performed. It emphasized the presence of the abnormal mass and its dimensions and location (Fig. 2).

**Fig. 3:**
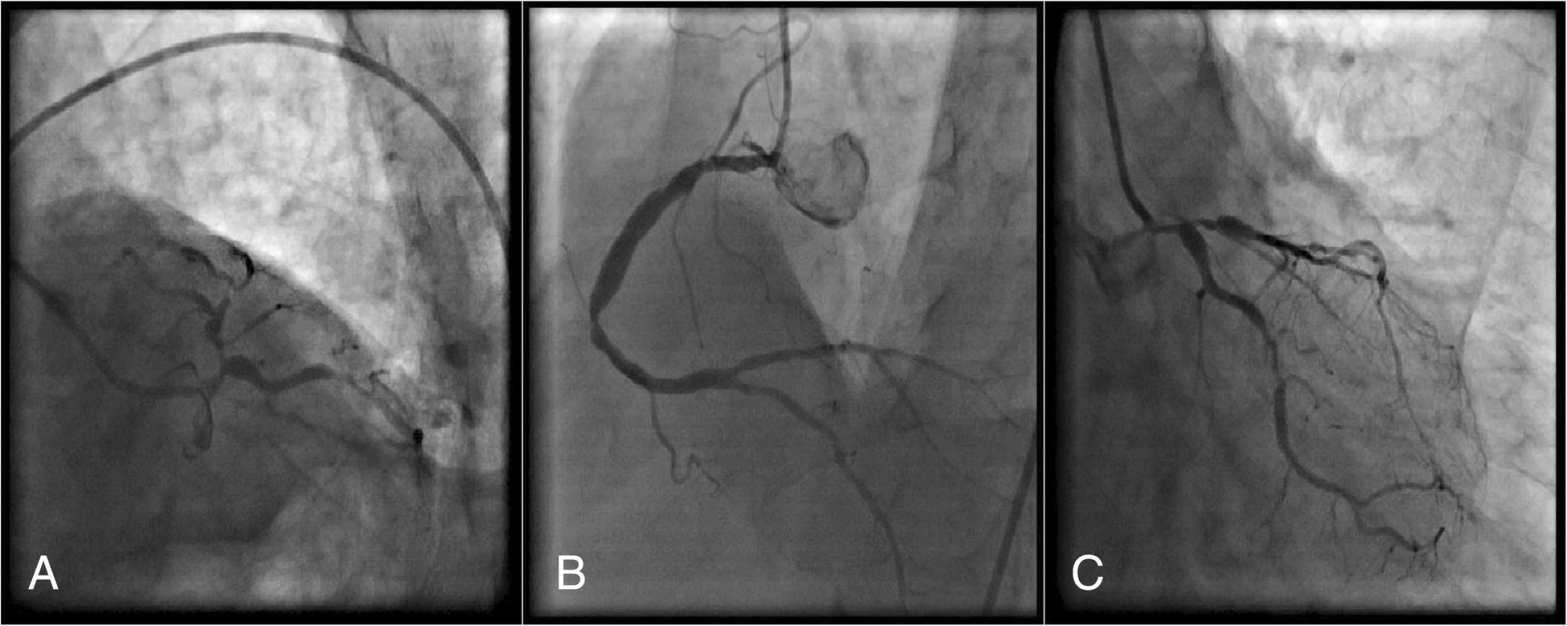
Coronary catheterization. **a** Spider view showed ostial left anterior descending lesion. **b** Multi-sequential right coronary artery lesions. **c** Right anterior oblique–caudal view showed severe stenosis in circumflex

He underwent coronary catheterization before the surgery. The angiography showed ostial lesion and severe stenosis in the mid left anterior descending (LAD) in addition to 80% stenosis in circumflex (CX), and the right coronary artery (RCA) had multi-sequential lesions beginning from the first segment (Fig. 3).

**Fig. 4:**
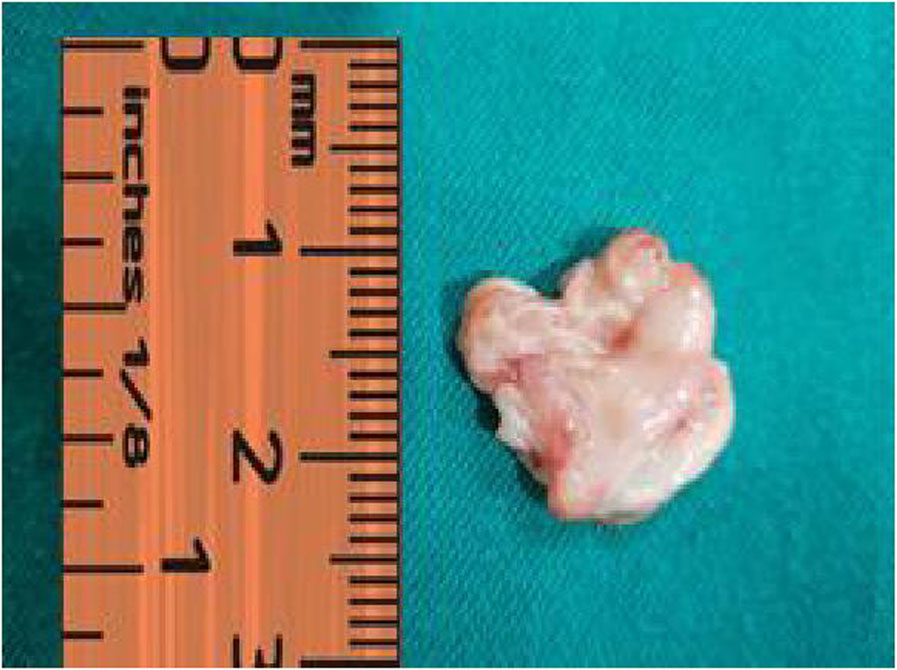
Gross view of the tissue after surgery

The surgery was delayed for a month due to a huge workload in the surgical department at that time. Since the case was rare and not fully understood by surgeons, it was classified as “cold case” in comparison to other on-list cases. Later on, our patient underwent coronary artery bypass graft (CABG) surgery and the abnormal tissue was surgically removed. The biopsy was sent to the pathology laboratory for further investigation (Fig. 4). The pathology report indicated a pure fibrous tissue with non-differentiated cells. Two weeks later, another TEE was done to assess the flow across the LVOT and the pressure gradient (PG) was normal. No residue of the abnormal tissue was seen. A timeline is shown in Table 1.

**Table 1:** Organized timeline

Three months earlier	Non-specific symptoms started (chest discomfort and exertional dyspnea)
Day 0	Visiting ER and admission to CCU
Day 1	Stabilizing vital signs and controlling symptoms/Management with metoprolol and O_2_
Day 2	TTE discovered a suspicious mass
Day 7	TEE emphasized the presence of accessory tissue
Day 14	Multi-slice CT studied the relation between the tissue and its neighboring anatomy and provided a primary impression of the coronary tree
Day 25	Coronary catheterization demonstrated multi-vessel disease with no associated anomaly
Day 40	CABG and surgical removal of the mass
Day 50	Biopsy showed fibrous tissue and TEE clarified normal PG across LVOT

*CABG* coronary artery bypass graft, *CCU* coronary care unit, *CT* computed tomography, *ER* emergency room, *LVOT* left ventricular outflow tract, *PG* pressure gradient, *TEE* transesophageal echocardiography, *TTE* transthoracic echocardiography

## Discussion

AMVT in adults is very rare and can mimic various causes of LVOT obstruction. While most cases are diagnosed in childhood, rarely do these cases happen in adulthood. Less than 30% of these anomalies occur in adults, two thirds of the patients were associated with other cardiac anomalies, and symptoms of LVOT obstruction developed early in the neonatal or childhood periods [1]. Although the underlying cause is still uncertain, it is suggested that AMVT results from an incomplete separation of the mitral valve from the endocardial cushion during cardiac development during embryogenesis [6]. Patients with AMVT anomaly may be asymptomatic but more frequently experience palpitations and fatigue [7].

Patients with AMVT become symptomatic when the mean gradient across the LVOT is more than 50 mmHg [2]. Severe obstruction is defined when LVOT gradient is over 50 mmHg, while the obstruction is defined as mild when the gradient is less than 31 mmHg [1]. AMVT has been variably described as sail-shaped, sac-like, balloon-like, parachute-like, and leaflet-like, or as a sheet, membrane, or pedunculated mass [8].

Despite the rarity of AMVT in adults, it should be kept in mind as part of the differential diagnosis of LVOT obstruction. Other LVOT masses that might cause obstruction include: myxoma, papillary fibroelastoma, and thrombus or vegetations, which can produce similar echocardiographic appearances. However, a detailed TTE and TEE can be used to differentiate them from AMVT [9]. Both TTE and TEE may help in the diagnosis, revealing possible associated lesions and complications [10]. Regarding treatment of this anomaly, cardiac surgery is indicated only in patients with significant LVOT gradients and those undergoing correction of other congenital cardiac defects in childhood or acquired disease if an adult. Surgery includes accessory tissue removal and, sometimes, artificial chordae implantation and annuloplasty, depending on the severity of the disease [11–13].

## Conclusion

Echocardiography is considered to be the cornerstone investigation tool when assessing an aortic valve stenosis regardless of the cause. Significant LVOT obstruction happens when the PG exceeds 50 mmHg through LVOT flow and surgery is the only treatment in this severe status. However, conservative therapy is indicated for PG less than 50 mmHg except when the lesion is associated with other congenital defects. Finally, when facing such a case in the future, it is recommended to make a more rapid surgical response.

## Additional files

Additional file 1: **Video S1.** Transthoracic echocardiography demonstrated left ventricle output obstruction caused by a mobile mass. (MP4 421 kb)

Additional file 2: **Video S2.** Transesophageal echocardiography showed the anatomical relation of the accessory tissue and the anterior leaflet of the mitral valve. (MP4 3570 kb)

## Abbreviations

AMVT: Accessory mitral valve tissue
bpm: Beats per minute
CABG: Coronary artery bypass graft
CX: Circumflex
LAD: Left anterior descending
LVOT: Left ventricular outflow tract
MS-CT: Multi-slice computed tomography
PG: Pressure gradient
RCA: Right coronary artery
TEE: Transesophageal echocardiography
TTE: Transthoracic echocardiography
